# Improvement of non-key traits in radiata pine breeding programme when long-term economic importance is uncertain

**DOI:** 10.1371/journal.pone.0177806

**Published:** 2017-05-18

**Authors:** Yongjun Li, Heidi Dungey, Alvin Yanchuk, Luis A. Apiolaza

**Affiliations:** 1 Scion (New Zealand Forest Research Institute), Rotorua, New Zealand; 2 Tree Improvement Branch, British Columbia Forest Service, Victoria, British Columbia, Canada; 3 School of Forestry, College of Engineering, University of Canterbury, Christchurch, New Zealand; Agresearch Grasslands Research Centre, NEW ZEALAND

## Abstract

Diameter at breast height (DBH), wood density (DEN) and predicted modulus of elasticity (PME) are considered as ‘key traits’ (KT) in the improvement in radiata pine breeding programmes in New Zealand. Any other traits which are also of interest to radiata pine breeders and forest growers are called ‘non-key traits’ (NKTs). External resin bleeding (ERB), internal checking (IC), number of heartwood rings (NHR) are three such non-key traits which affect wood quality of radiata pine timber. Economic importance of the KTs and NKTs is hard to define in radiata pine breeding programmes due to long rotation period. Desired-gain index (DGIs) and robust selection were proposed to incorporate NKTs into radiata pine breeding programme in order to deal with the uncertainty of economic importance. Four desired-gain indices A-D were proposed in this study. The desired-gain index A (DGI-A) emphasized growth and led to small decrease in ERB and small increase in IC and NHR. The expected genetic gains of all traits in the desired-gain index B (DGI-B) were in the favourable directions (positive genetic gains in the key traits and negative genetic gains in the non-key traits). The desired-gain index C (DGI-C) placed emphasis on wood density, leading to favourable genetic gain in the NKTs but reduced genetic gains for DBH and PME. The desired-gain index D (DGI-D) exerted a bit more emphasis on the non-key traits, leading large favourable reduction in the non-key traits and lower increase in the key traits compared with the other DGIs. When selecting both the key traits and the non-key traits, the average EBVs of six traits were all in the same directions as the expected genetic gains except for DBH in the DGI-D. When the key traits were measured and selected, internal checking always had a negative (favourable) genetic gain but ERB and NHR had unfavourable genetic gain in the most of time. After removing some individuals with high sensitivity to the change of economic weights, robust desired-gain selection made genetic gains of all the key and non-key traits to move a little bit toward unfavourable directions in the four indices. It is concluded that desired-gain index combined with robust selection concept is an efficient way for selecting the key and non-key traits in radiata pine breeding programmes.

## Introduction

Radiata pine is the dominant species of plantation forests in New Zealand. Its wood is used as structural and appearance timber and in manufacture of various products like boards, veneers, panel products, pulp and paper. The three ‘key traits’ for the improvement of radiata pine are growth, wood density and stiffness as they are currently considered the most economically important to the New Zealand forest industry. Any other traits which are also of interest to radiata pine breeders and forest growers are called ‘non-key’ traits (NKTs). External resin bleeding, internal checking, heartwood content are three such non-key traits which affect wood quality of radiata pine timber.

External resin bleeding and internal checking are two wood defects in radiata pine and lower the value of appearance-grade timber. External signs of resin bleeding on the stem may indicate the presence of internal resin pockets [1, 2]. Resin causes poor paint adhesion and bleed from beneath the painted surface in treated products (Mackie pers. Comm., 2013). Internal checking, also called intra-ring checking, is particularly prominent in the butt log. It usually does not appear until after drying and is not visible on the surface of the wood [3]. The earlywood has thin cell walls and collapses more than the latewood and the resulting tensile force in the earlywood causes splitting (internal checking). Internal checking appears to be worse on sites exposed to drought, waterlogging or frost [3]. Internal checking is manifest in appearance products, particularly finger-jointed defect-free boards and clears for internal and external use. For appearance-grade products there is no tolerance to resin and internal checking. Reducing incidence of internal checking and resin bleeding for radiata pine would ensure the reliability and quality required to export markets. Heartwood in radiata pine has some undesirable features that can cause structural weakness and warping due to its dark appearance, low durability [4] and the fact that it is difficult to treat chemically [5]. The economic value of stem wood will be increased by reducing the content of heartwood.

To incorporate NKTs into radiata pine breeding programme, a multiple-trait selection method needs to be used to improve the KTs and sustain NKTs simultaneously. An index selection has been considered as an efficient way to select multiple traits in plant and livestock breeding programmes [6]. Index selection relies on a selection index that combines all traits of importance [7]. The advantage of index selection is that genetic improvement is expressed in net merit and that strength in one trait can make up for relative weakness in another trait. Typically, it is implemented when breeding objective traits, selection criteria and economic weights are all clearly defined. These traits are weighted using economic weights, to create an index for selection. Selection criteria that are used for ranking selection candidates should be heritable and correlated to the breeding objective traits. An economic weight represents the discounted profit of one unit improvement of a trait without altering other traits presented in the objective [8]. The economic weight of a trait is determined by analysing how a marginal change in the objective traits affects the economic or biological outcome in the production system [9–14]; hence, the use of a selection index maximises economic output for a given set of economic weights on traits included in selection. The efficiency of an index selection depends on the accurate definition of the economic weights of the breeding objective traits and on the quality of the genetic parameters and breeding values [15–17].

In tree breeding, the economic weights for breeding objective traits might not be quantified or defined accurately due to long rotation cycle, and the poor connection between tree traits and final products. For example, radiata pine has a rotation cycle of 25–30 years [18] and obviously it is very difficult to define economic weights for next two or three generations. Therefore, some uncertainty exits in the definition of economic weights for tree breeding. Desired-gain index can be used when economic weights are unknown, unimportant or difficult to assess [19]. With desired-gain index, breeders’ primary concern is the genetic changes of targeted traits rather than economic output and the economic weights of breeding objective traits are not needed [20]. With the desired-gain index, breeders are able to specify the relative amount of improvement they would desire to achieve in each trait included in the selection index [20–22]. A set of weighting coefficients can be found which produces predicted gains in individual traits in the selection index that are proportion to the desired-gains specified by breeders [23]. Evison and Apiolaza [24] proposed a robust selection to deal with the uncertainty of economic weights and reduce selection risk in forest tree breeding by excluding candidates that are highly sensitive to the changes of economic weights.

In this study, a number of desired-gain selection indices were developed to incorporate NKTs in radiata pine breeding programmes for selecting the KTs and NKTs simultaneously. The robust selection concept was incorporated with the desired-gain selection indices to deal with the uncertainty of economic importance of traits.

## Materials and methods

### Genetic material

Data from a Female Tester trial series, established between 1992 and 1993 with 293 pollen parents crossed with each of five female testers with mid-to-high growth and form ranking and tested at two sites: Esk Forest in Hawkes Bay (39° 15’ S, 176° 42’ E) and Woodhill Forest in Northland (36° 46’ S, 174° 21’ E), were used in this study. The trial series comprised of 948 families, each having an average of 9.45 progeny across two sites. The total number of progeny in this trial series was 8,959. The Female Tester trial had a sets-in-reps single-tree-plot experimental design with 30 replicates.

### Trait assessment

Assessments of diameter-at-breast-height (DBH), wood density (DEN), predicted modulus of elasticity (PME), internal checking (IC), external resin bleeding (ERB) and number of heartwood rings (NHR) were available in the Female Tester trial series. DBH was assessed at 9 years of age at both sites. Wood density was assessed as basic density at Esk only using 5 mm pith-to-bark breast-height increment core at 15 years of age. The basic density of increment core was determined as the sample weight at 4.5% moisture content divided by the volume of the sample measured green using the water displacement method [25]. Internal checking was assessed at 13 years of age at Esk and at 14 years of age at Woodhill using a scale of 0–3: 0 = none, 1 = slight, 2 = moderate and 3 = severe. ERB severity score was assessed at 9 years of age at Esk and at 10 years of age at Woodhill on a scale of 1–4: 1 = none, 2 = low, 3 = moderate and 4 = severe. Heartwood content was assessed on a 5 mm pith-to-bark increment core extracted at breast height from each sample tree at 16 years of age at Esk and at 17 years of age at Wood hill. The number of heartwood rings of each core was measured. PME was assessed using standing tree acoustic velocity at 9 years of age at Esk and at 12 years at Woodhill. PME was estimated as the product of green wood density and the square of acoustic velocity using TreeTap, designed by Dr Michael Hayes, University of Canterbury [26]. The acoustic velocity was assessed as the transit time between two transducers (one generates, the other receives the wave) set at a known distance on the tree stem. Table 1 shows a summary statistics of phenotypes assessed for DBH, DEN, PME, IC, ERB and NHR at two sites.

**Table 1:** Summary statistics of phenotypes assessed: Diameter-at-breast-height (DBH), wood density (DEN), predicted modulus of elasticity (PME), internal checking (IC), external resin bleeding (ERB), number of heartwood rings (NHR).

Site	Trait	No of observations	Mean	SD	Min	Max	Assessment age	Unit
Esk	DBH	4702	248.44	29.93	101.00	356.00	9	mm
	DEN	1525	343.05	20.49	279.00	421.00	15	kg/m^3^
	PME	1681	3.116	0.62	1.54	5.73	9	GPa
	IC	1089	0.90	0.92	0.00	3.00	13	score
	ERB	5091	2.60	0.80	1.00	4.00	9	Score
	NHR	3315	3.46	1.02	1.00	9.00	16	Number of rings
Woodhill	DBH	2529	196.49	32.20	100.00	326.00	9	mm
	DEN ^†^	-	-	-	-	-	-	-
	PME	1329	9.89	1.97	4.27	17.22	12	GPa
	IC	1741	0.85	0.81	0.00	3.00	14	score
	ERB	2522	2.52	0.82	1.00	4.00	10	Score
	NHR	1682	3.65	1.00	1.00	8.00	17	Number of rings

^**†**^ Phenotype was not available at this site.

### Genetic analysis

Variance-covariance estimation among six key and non-key traits described above was conducted with a multivariate analysis with individual tree linear mixed models, assuming common additive and residual variance across sites implemented with ASReml [27]:

$$y=Xb+Z_{s}s+Z_{a}a+e$$ (1)

where ***y*** is a vector of observations, ***b*** is a vector of fixed effects (i.e. population mean, site and replicate), ***s*** is a vector of random effects of sets within replicate, ***a*** is a vector of random additive genetic effects of individual genotypes with a distribution of

$$a\sim N\left(0,G⊗A\right),\text{ where }G=\left[\begin{array}{ccc} \sigma_{a_{1}}^{2} & \cdots & \sigma_{a_{1}a_{6}}\\ \vdots & \ddots & \vdots \\ \sigma_{a_{1}a_{6}} & \cdots & \sigma_{a_{6}}^{2} \end{array}\right],$$ (2)

where $\sigma_{a_{1}}^{2},\cdots ,\sigma_{6}^{2}$ are the additive genetic variances of traits 1 to 6, and $\sigma_{a_{i}a_{j}}$is the additive genetic covariance between trait *i* and trait *j*, *i* = 1 to 6 and *j* = 1 to 6, ***A*** is the numerator relationship matrix generated from the pedigree; and ***e*** is a vector of random residual effects with a distribution of

$$e\sim N\left(0,E⊗I\right),\text{where }E=\left[\begin{array}{ccc} \sigma_{e_{1}}^{2} & \cdots & \sigma_{e_{1}e_{6}}\\ \vdots & \ddots & \vdots \\ \sigma_{e_{1}e_{6}} & \cdots & \sigma_{e_{6}}^{2} \end{array}\right]$$ (3)

where $\sigma_{e_{1}}^{2},\cdots ,\sigma_{6}^{2}$ are the residual variances of traits 1 to 6, and $\sigma_{e_{i}e_{j}}$ is the residual covariance between trait *i* and trait *j*, *i* = 1 to 6 and *j* = 1 to 6, ***I*** is the identity matrix. ***X*, *Z***_***s***_ and ***Z***_***a***_ are the incidence matrices associating phenotypes with fixed and random effects of ***b***, ***s*** and ***a***, respectively.

Narrow-sense heritability of trait *i* was calculated as

$$h_{i}^{2}=\frac{\sigma_{a_{i}}^{2}}{\sigma_{a_{i}}^{2}+\sigma_{e_{i}}^{2}}$$ (4)

where $\sigma_{a_{i}}^{2}$ and $\sigma_{e_{i}}^{2}$ are the estimated additive genetic variance and the estimated residual variance for trait *i*, respectively. Genetic correlation between trait *i* and trait *j* was calculated as

$$r_{g_{ij}}=\frac{\sigma_{a_{ij}}}{\sqrt{\sigma_{a_{i}}^{2}\cdot \sigma_{a_{j}}^{2}}}$$ (5)

where $\sigma_{a_{ij}}$is the estimated additive genetic covariance between trait *i* and trait *j*, $\sigma_{a_{i}}^{2}$ is the estimated additive genetic variance for trait *i*, and $\sigma_{a_{j}}^{2}$ is the estimated additive genetic variance for trait *j*. Phenotypic correlation between trait *i* and trait *j* was calculated as

$$r_{p_{ij}}=\frac{\sigma_{a_{ij}}+\sigma_{e_{ij}}}{\sqrt{\left(\sigma_{a_{i}}^{2}+\sigma_{e_{i}}^{2}\right)(\sigma_{a_{j}}^{2}+\sigma_{e_{j}}^{2}})}$$ (6)

### Determining breeding objectives

The breeding objective or an aggregate economic genotype was developed by Hazel [8] as a linear combination of additive genetic values of two or more traits weighted by their relative economic values:

$$H=v_{1}g_{1}+v_{2}g_{n}+\;\cdots +v_{n}g_{n}=v^{\prime }g$$ (7)

where ***v***^*′*^ ***=* [***v*_*1*_,*v*_*2*_,*…*,*v*_*n*_] is the vector of relative economic weights and ***g***^*′*^ ***=* [***g*_*1*_,*g*_*2*_,*…*,*g*_*n*_] is the vector of additive genetic values. A selection index (*I*) which maximizes the correlation between *I* and *H* was calculated as

$$I=b_{1}{\hat{g}}_{1}+b_{2}{\hat{g}}_{2}+\;\cdots +b_{m}{\hat{g}}_{m}=b^{\prime }\hat{g}$$ (8)

where ***b***^***′***^ ***=* [*b***_*1*_,*b*_***2***_,*…*,*b*_***m***_] is the vector of index weights and ${\hat{g}}^{\prime }=[{\hat{g}}_{1},{\hat{g}}_{2},\ldots ,{\hat{g}}_{m}]$ is the vector of selection criteria or vector of EBVs. In this study, DBH, DEN, PME, IC, ERB and NHR are both selection criteria and breeding objective traits. Index weight was calculated [28] as

$$b=P^{-1}Gv$$ (9)

where ***P*** is the genetic variance-covariance matrix among the traits in selection criteria and ***G*** is the genetic variance-covariance matrix between the traits in selection criteria and the traits in the breeding objectives.

The variance of the selection index,$\;\sigma_{I}^{2}$, was derived as $\sigma_{I}^{2}=b^{\prime }Pb$. The expected genetic gain in the additive genetic values of the *j*^*th*^ trait in the aggregate genotype due to selection on the index (Δ*G*_*j*_) were estimated as

$$\Delta G_{j}=i\frac{b^{\prime }G_{j}}{\sigma_{I}}$$ (10)

where *G*_*j*_ denotes the *j*^*th*^ column of ***G*** and *i* is the selection intensity, which was equal to 2.063 for selection of the top 5% of candidates in the current study.

### Derivation of pseudo-economic weights for a desired-gain index

In the desired-gain index derived, expected genetic gain for each trait was pre-defined according to long-term targets of breeding programme. Suppose breeders want to change the means of the six KTs and NKTs by the amount of

$$\Delta G_{T}=\left[\Delta G_{dbh},\;\Delta G_{den},\;\Delta G_{pme},\Delta G_{ic},\Delta G_{erb},\Delta G_{nhr}\right],$$ (11)

for example,

$$\Delta G_{T}=\left[2,\;20,0.77,\;-0.30,\;-0.25,\;-0.10\right].$$ (12)

A set of the optimal pseudo-economic weights which provided the pre-determined “desired” genetic gains were found with an optimization algorithm—differential evolution (DE) algorithm [29]. The DE objective function (*f*) was defined as

$$f=\sum_{i}^{6}{\left(\Delta G_{i}-\Delta G_{T_{i}}\right)}^{2}$$ (13)

where Δ*G*_*i*_ is the expected genetic gain for trait *i* from index selection calculated using Eq 10 and $\Delta G_{T_{i}}$ is the targeted genetic gain for trait *i*. The DE used a population with 20 individuals to evolve and identify the minimum value of the objective function. Each DE individual was represented by a ‘chromosome with 5 loci’, with the value at each locus representing the economic weights for DEN, PME, IC, ERB and NHR respectively. Thus, each DE individual or chromosome represented a possible set of pseudo-economic weights. Expected genetic gains of the key and non-key traits were estimated using Eq 10 for each DE individual, and the objective function was calculated with Eq 13. In the founder population of the DE, values at each locus were generated from a uniform [0,1] distribution. To evolve the population, in the next generation of the DE, the value at locus *j* of chromosome *i* (*V*_*ij*_) had a range of 0 and 1, being assigned a new value $\left(V_{ij}^{\prime }\right)$ if a uniform random number was less than the crossover rate of 0.8,

$$V_{ij}^{\text{'}}=2.5\left(V_{aj}-V_{bj}\right)+V_{cj}$$ (14)

where *V*_*aj*_, *V*_*bj*_, and *V*_*cj*_ are the values for locus j on chromosomes *a*, *b* and *c* from the previous generation (*a*, *b* and *c* were randomly chosen and *a* ≠ *b* ≠ *c* ≠ *i*). For the whole chromosome *i*, the new values, $V_{ij}^{\prime }$ replaced *V*_*ij*_ if the objective function of $V_{ij}^{\prime }$ was higher than that of *V*_*ij*_, otherwise *V*_*ij*_ remained unchanged. For each generation of the DE, the chromosome with the highest objective function was taken as the solution for that generation of the DE. The DE was considered to have converged when the objective function of the best solution changed by less than 0.0001 within 200 generations. This typically occurred within 20000 generations of the DE. Upon convergence, individuals selected from the chromosome with the lowest objective function were selected as the final solutions for deriving the value of the economic weights for the KTs and NKTs.

### Definition of desired-gain indices

Four desired-gain selection indices were compared in this study (Table 2). Targeted genetic gains per generation for the key traits and non-key traits defined in Table 2 were arbitrary values in the directions that New Zealand radiata pine breeders aspired. The actual values of targeted genetic gain may be not reached in the final selection indices. The index A (DGI-A) aimed to maximize growth with targeted genetic gains of 5 mm, 10 kg/m^3^, 0.77 GPa, -0.30, -0.25 and -0.10 DBH, DEN, PME, IC, ERB and NHR, respectively. The index B (DGI-B) aimed at obtaining favourable genetic gains for both the key and non-key traits with targeted genetic gains of 2 mm for DBH, 20 kg/m^3^ for DEN and remained the same for other traits as those in the DGI-A. The index C (DGI-C) targeted high density with targeted genetic gains of 0 mm for DBH and 36 kg/m^3^ for DEN and remained the same for other traits as those in the DGI-B. The index D (DGI-D) targeted a low gain in the key traits and a high reduction of the incidence for the non-key traits. The pseudo-economic weights of the key and non-key traits were found using the differential evolution algorithm. Here we used relative economic weights for breeding objective traits. An arbitrary economic weight for DBH was fixed to 100 and the economic weights for the other objective traits were varied, being relative to that for DBH.

**Table 2:** Targeted genetic gains for diameter-at-breast-height (DBH), wood density (DEN), predicted modulus of elasticity (PME), internal checking (IC), external resin bleeding (ERB), number of heartwood rings (NHR) in four desired-gain selection indices: A-D.

Selection index	DBH	DEN	PME	IC	ERB	NHR
A	5	10	0.77	-0.3	-0.25	-0.1
B	2.5	20	0.77	-0.3	-0.25	-0.1
C	0	36	0.77	-0.3	-0.25	-0.1
D	0	10	0.37	-0.9	-0.9	-0.9

There were 8,959 selection candidates in the Female Tester series in this study. Selection of the top 5% out of 8,959 selection candidates was made using selection indices derived from Eq 9 and the pseudo-economic weights obtained in the desired gain indices A-D. The average EBVs of the key and non-key traits of the selected individuals were used as a standard approach for the estimation of genetic gains.

### Robust desired-gain selection

**Fig 1:**
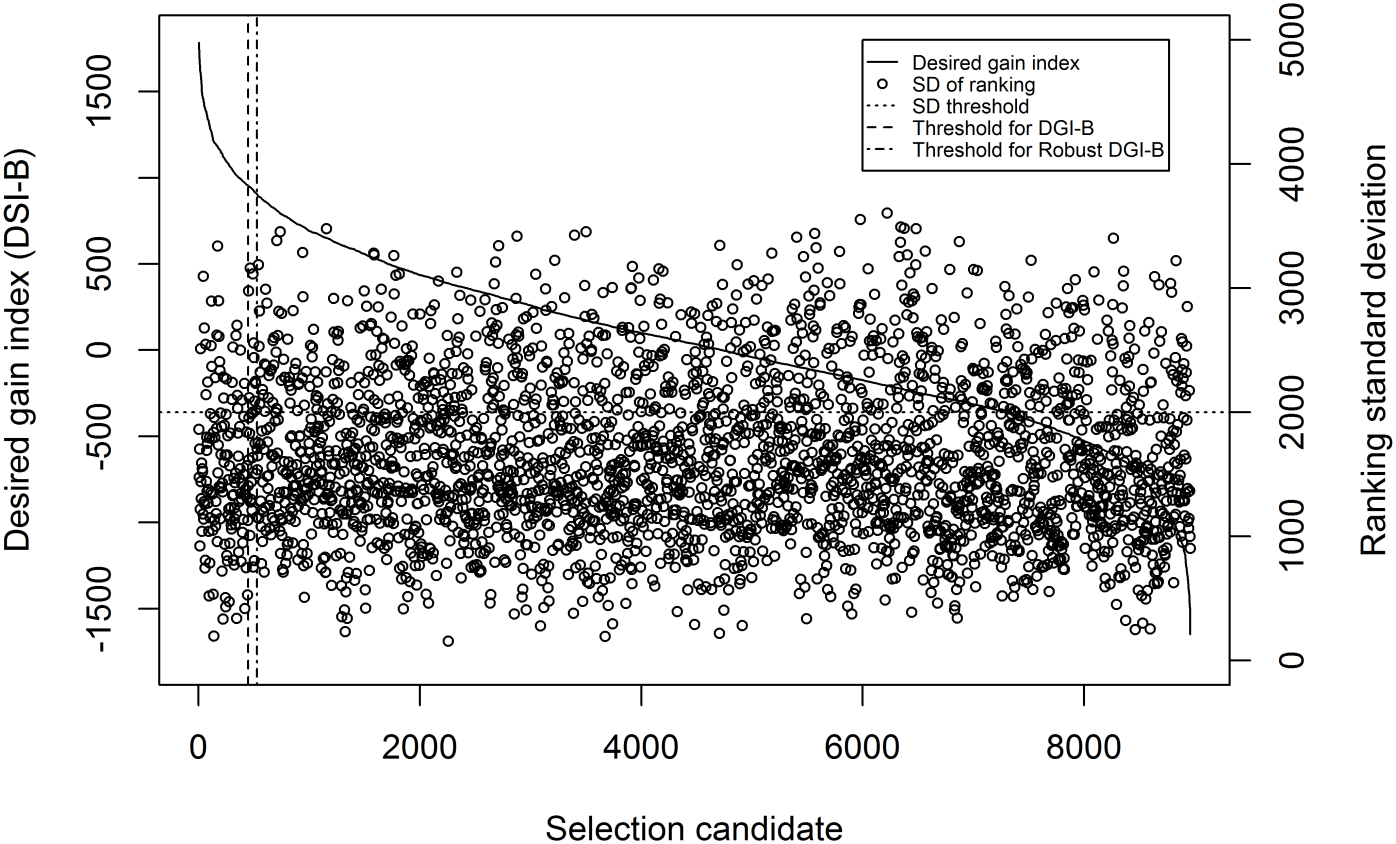
A diagram of index DGI-B and standard deviation of ranking for selection candidates. The selection candidates were sorted by DGI-B from the highest to the lowest. Standard deviation of ranking was calculated from 1000 rankings when economic weights of the key traits were sampled from a triangular distribution for 1000 times. Desired-gain selection selected the individuals on left of the threshold for DGI-B and Robust DGI-B selected those on left of the threshold but excluded the individuals with high ranking standard deviation.

Robust selection is a method proposed for dealing with the uncertainty of economic weights [24]. Economic weights for DBH, DEN, PME, IC, ERB and NHR were sampled from a triangular distribution with its minimum, median and maximum of 1, 100 and 200 for DBH, 1, 20 and 40 for DEN, and 1, 2500 and 5000 for PME, -1000, -500, 0 for IC, -200, -100 and 0 for ERB and -2000, -1000 and 0 for NHR. Selection index weights were calculated using Eq 9 and an index was derived for the selection candidates. The selection candidates were ranked by the index. Economic weights were sampled 1,000 times and there were therefore 1,000 rankings for the selection candidates. The standard deviation of the rankings was calculated. The standard deviation of rankings demonstrated sensitivity of individuals to the change of economic weights of the KTs and NKTs. Selection was made based on the desired-gain index but excluded the individuals with a ranking standard deviation of above 2000. The average EBVs of selected individuals were compared between the desired-gain index selection and the robust desired-gain index selection. Fig 1 shows an example of desired-gain index selection and robust desired-gain index selection.

## Results

### Genetic parameters of the non-key and key traits

Table 3 shows heritabilities, genetic variance and genetic correlations for the key and non-key traits estimated across sites. We estimated a high heritability in wood density (h^2^ = 0.67) and a low heritability in DBH (h^2^ = 0.08). The genetic correlation between DBH and DEN was -0.37. The genetic correlation between DBH and ERB was 0.33 and that between DBH and NHR was 0.45. The genetic correlation between DEN and PME was 0.24 and that between DEN and IC was -0.53.

**Table 3:** Heritabilities (h^2^), genetic variance $\left(\sigma_{a}^{2}\right)$, and genetic correlations for diameter-at-breast-height (DBH), wood density (DEN), predicted modulus of elasticity (PME), internal checking (IC), external resin bleeding (ERB), number of heartwood rings (NHR), estimated across the two sites: Woodhill and Esk.

Trait	h^2^	$\sigma_{a}^{2}$	Genetic correlation				
			DBH	DEN	PME	IC	ERB
DBH	0.08	81.24					
DEN	0.67	339.60	-0.37				
PME	0.28	0.51	-0.27	0.24			
IC	0.44	0.31	0.25	-0.53	-0.32		
ERB	0.32	0.21	0.33	-0.31	-0.11	0.21	
NHR	0.24	0.23	0.45	-0.08	-0.18	-0.19	0.22

### Expected genetic gain from different desired-gain indices

Index weights and expected genetic gains for DBH, DEN and PME in DGIs A-C are shown in Table 4. The desired-gain index A (DGI-A), emphasizing growth, resulted in a small decrease (favourable changes) in IC and ERB and a small increase (unfavourable changes) in NHR. The expected genetic gains of all traits achieved in the DGI-B were in the favourable directions (positive genetic gains in the key traits and negative genetic gains in the non-key traits). The DGI-C, emphasizing on wood density, resulted in favourable genetic gains in the NKTs but resulted in negative (unfavourable) genetic gains for DBH and PME. The DGI-D, which exerted some emphasis on the NKTs, led to a large reduction in the NKTs, a bigger increase in PME, a lower increase in DBH and DEN, compared with the genetic gains achieved in the DGI-A and DGI-B.

**Table 4:** Index weights and expected genetic gains for diameter-at-breast-height (DBH), wood density (DEN), predicted modulus of elasticity (PME), internal checking (IC), external resin bleeding (ERB), number of heartwood rings (NHR) in four desired-gain selection indices.

Selection index	DBH	DEN	PME	IC	ERB	NHR
**Index weight**						
A	11.51	7.44	102.34	145.35	-65.92	66.20
B	17.05	16.88	144.41	111.30	-158.40	-30.47
C	22.74	30.77	-16.79	295.42	-300.91	-37.95
D	46.13	9.427	1044.08	-440.15	-388.47	-783.793
**Expected genetic gain** (units are the same as those in Table 1)						
A	4.66	9.96	0.32	-0.01	-0.06	0.11
B	2.44	19.99	0.54	-0.22	-0.23	-0.02
C	-0.16	36	0.22	-0.26	-0.41	-0.05
D	0.00	10.23	0.85	-0.32	-0.18	-0.19

### Performance of selected individuals

Average EBVs of selected individuals for the traits were calculated for desired-gain index selection when selecting both the KTs and NKTs or selecting the KTs only (Table 5). When selecting both the KTs and NKTs, the average EBVs of six traits were all in the same directions as the expected genetic gains which are listed in Table 4, except that the expected genetic gain for DBH was 0 and the average EBVs for DBH was -0.65 in the DGI-D. DGI-A led to lower performance in DEN and a positive (unfavourable) genetic gain in NHR. DGI-C led to a small increase of genetic gain for DBH and large decrease and favourable genetic gain in the NKTs. When the NKTs were not selected, IC always had a negative (favourable) genetic gain but ERB and NHR had unfavourable genetic gain in the most of time. After removing some individuals with high sensitivity to the change of economic weights, robust desired-gain index selection made genetic gains of all the KTs and NKTs to move a little bit toward unfavourable directions in the four indices when selecting both the KTs and NKTs (DGIs A-D) (Table 6).

**Table 5:** Average EBVs of selected individuals for diameter-at-breast-height (DBH), wood density (DEN), predicted modulus of elasticity (PME), internal checking (IC), external resin bleeding (ERB) and number heartwood rings (NHR in desired-gain indices DGIs A-D when selecting both the KTs and NKTs or selecting the KTs only.

Scheme	DBH	DEN	PME	IC	ERB	NHR
**Selection on both key traits and non-key traits**						
A	3.37	12.25	0.59	-0.18	-0.07	0.11
B	0.07	18.04	0.63	-0.38	-0.21	-0.01
C	-2.95	20.33	0.21	-0.26	-0.32	-0.12
D	-0.65	16.17	0.80	-0.39	-0.16	-0.07
**Selection on key traits only**						
A	5.87	6.04	0.72	-0.38	0.06	0.28
B	3.61	10.26	0.81	-0.42	0.00	0.19
C	-1.53	20.88	0.45	-0.45	-0.21	0.01
D	5.59	3.74	0.78	-0.36	0.10	0.25

**Table 6:** Average EBVs of selected individuals for diameter-at-breast-height (DBH), wood density (DEN), predicted modulus of elasticity (PME), internal checking (IC), external resin bleeding (ERB) and number heartwood rings (NHR) in the robust desired-gain indices DGIs A-D when selecting both the KTs and NKTs.

Scheme	DBH	DEN	PME	IC	ERB	NHR
A	3.36	11.58	0.55	-0.17	-0.05	0.11
B	-0.11	17.42	0.62	-0.37	-0.20	0.00
C	-2.70	19.20	0.20	-0.23	-0.31	-0.12
D	-0.61	15.39	0.77	-0.38	-0.16	-0.06

## Discussion

Genetic correlations between the non-key traits and the key traits have been reported in the literature. Internal checking had negative genetic correlations (-0.28 to -0.59) with wood density [30–32], which was favourable for reducing the incidence of internal checking while increasing wood density. The genetic correlation between internal checking and growth varied in the literature. A negative genetic correlation (-0.13 to -0.33) between internal checking and DBH was reported by [30, 31] whereas a positive genetic correlation (0.25) between them was reported by [32]. Genetic correlations between internal checking and stiffness were negative (-0.07 to -0.21) [32] but no relationship between them has been reported [30, 31]. ERB had a high positive genetic correlation with DBH (0.54), low positive genetic correlation with wood density (0.12) and a negative genetic correlation with stiffness (-0.25) [31] but it had no genetic correlation with DBH, wood density and stiffness [30]. Genetic correlation between growth and number of hardwood rings was high (0.38–0.42) at Woodhill and Esk and low (-0.06 to 0.16) at Phoenix and Bobcat [5]. In the current study, DBH had positive genetic correlations with internal checking, external resin bleeding and number of hardwood rings, unfavourable for increasing growth and reducing incidence of non-key traits. Wood density and stiffness had negative genetic correlations with the non-key traits, which was favourable genetic correlations for increasing wood density and stiffness and reducing incidence of the non-key traits.

Heritability estimates were 0.33 to 0.38 for ERB, 0.11 to 0.35 for IC and 0.11 to 0.44 for NHR [5, 30–32]. The average heritability was 0.21 for DBH and 0.49 for PME in the literature [33]. The heritabilities for IC, ERB and NHR in the current study were similar to or higher than those reported in the literature while the heritabilities for DBH and PME in the current study was lower than the average heritabilities reported in the literature. In the statistical analysis of this study, a multi-variate model was used to estimate heritabilities of traits and genetic correlations between them. The phenotypes were assessed at two sites for all traits except wood density. Genotype by environment interactions for each trait were accounted for in the genetic model. The Site-site genetic correlations estimated were above 0.8 for IC, ERB and NHR and below 0.7 for DBH and PME (results were not shown). DBH and PME were heavily affected by genotype by environment interaction, which might be the reason why low heritabilities were observed for DBH and PME and low genetic correlations between PME and DBH and between PME and DEN in the current study.

High expected gains for DEN, PME and IC were achieved in most of selection indices tested. This might be because of the high heritability for DEN and the moderate favourable genetic correlations for PME and IC. Of the all the selection schemes examined in this paper, expected genetic gains for PME and IC were all in the favourable directions, which was verified by the average EBVs of selected individuals from the Female Tester experiment and by the simulation results. When selecting the NKTs, the average EBV of IC was high enough to reduce its incidence in the population. Therefore, internal checking would reduce in the population even where no selection is applied, based on the Female Tester trial series- unless also selecting for high DEN.

Lower expected genetic gains for three traits, DBH, ERB and NHR, were the consequence of low heritabilities in DBH, unfavourable genetic correlations between DBH and DEN, and unfavourable genetic correlations among DBH, ERB and NHR. The results in this study show that ERB and NHR should be selected in the breeding programme otherwise they will increase while increasing for DEN and DBH. Due to the high genetic correlation between DBH and NHR, it was very hard to select for increasing growth while at the same time, decreasing NHR. If the genetic gain for DBH was 3.37 mm, the genetic gain of NHR was found to be positive in unfavourable direction (DGI-A in Table 5). Or a selection (DGI-C), which lead to a decrease of more than 0.12 in NHR, resulted in a negative genetic gain in DBH (-2.29mm) (Table 5). A restricted selection index may be useful to restrict genetic gain in NHR to zero while increasing growth rate or to restrict genetic gain in growth to zero while reducing heartwood content [34, 35]. When expecting a low genetic gain in growth, ERB and NHR, there is not much we can do to improve this situation because there must be unknown and strong physiological relationships between these traits. However, one thing we can do is to collect more data to obtain more accurate estimation of heritabilities and variance-covariance matrix of traits involved. At that stage, we may not have more room to increase growth and reduce ERB and NHR simultaneously in the whole population. But if we can find correlation breakers, we may use clonally deployed correlation breakers to achieve this.

This study found that there was little room for reducing the incidences of the NKTs while increasing genetic gain in growth, wood density and stiffness. Breeders, however, can still find a selection index which leads to favourable genetic gains for all traits, for example, desired-gain index B (DGI-B). However, which selection index is the best index for a breeder depends on the overall breeding objective of the breeder and the end-user of the crop.

This study used a differential evolution algorithm to find out the optimal pseudo-economic weights of growth, wood density and stiffness for predetermined expected genetic gains for both the KTs and the NKTs, when exact economic weights for breeding objective traits were not available or unsure in the long-term. This algorithm allows breeders to predetermine desired-gains for all traits and find the optimal pseudo-economic weights. In Table 4, the expected genetic gain from the desired-gain selection schemes were not exactly as the same as the predetermined targeted genetic gains (Table 2), even though a genetic gain close to the targeted number was found. Two possible reasons could explain the differences. One is that the predetermined targeted genetic gain might be biologically impossible to obtain, based on the relationship among traits. Another reason could be the inefficiency of differential evolution algorithms, which means that differential genetic algorithms did not find the global minima of the objective function. Further improvement of the differential evolution algorithms may be needed.

Formal economic weights has been estimated for eucalypts [12], radiata pine [14, 36, 37] and Scots pine [38] but the use of economic weights in forest tree breeding has not been fully implemented [39]. possible reason is the uncertainty of economic weights [39]. Economic values of the objective traits in a breeding programme are realised after several decades in the future. Even for relatively fast-growing tree species such as radiata pine, the lag between breeding decision and realisation of value from harvested trees can be 50 years or more [24]. To mitigate the effects of uncertainty, one option is to develop several breeding populations, with different breeding objectives, which then reduces potential losses caused by erroneous predictions about future industrial structures. Another option is to merge several breeding objectives into one generic breeding objective by weighing the breeding objectives according to the expected relative importance of different industry sectors or by using methods to make trade-offs depending on risk perception [40]. Evison and Apiolaza (24) proposed a robust selection to remove individuals with high standard error of ranking change when different economic weights applied. The current study provided one more option to deal with the uncertainty of economic weights for species with long rotation. Robust desired-gain index, a combination of desired-gain index and robust selection, leads to a good outcomes for the KTs and the NKTs in radiata pine breeding. The implication of the robust desired-gain index is that we do not need to derive economic weights for breeding objective traits as long as we know the direction of improvement of traits.

Robust selection excluded individuals with high sensitivity to the change of economic weights [24]. It also reduced genetic gains in all traits because the individuals which were sensitive to the change of economic weights had high performance in the KTs or low incidence in the NKTs. In this study, an arbitrary threshold was used. Breeders can determine the magnitude of threshold for ranking standard deviation used by themselves. The lower the threshold, the more reduction of genetic gain in the key traits or the less reduction of the non-key traits would be expected.

With the development of large-scale genotyping techniques, genomic selection is being increasingly used as a major tool for selection in forest tree breeding [41–48]. Genomic selection can be undertaken well before the normal age of phenotyping, e.g. around age 8 in radiata pine. DNA can be extracted and genotyping undertaken on only a few needles, before the age of six months. Generation intervals for breeding can be greatly reduced and the expected genetic gains per unit of time can be increased [43]. The selection index used in the Eq 8 was derived from using the estimated breeding values of selection criteria. The desired-gain index and robust selection can be easily extended to use genomic estimated breeding values that are derived from genomic selection [49–52]. If there are not estimated breeding values available, phenotypic assessment can be used to derive robust desired-gain index by using the method in Kennedy, Yanchuk [53].

## Conclusions

There were favourable genetic correlations between DEN, PME and IC and a high heritability in DEN in the Female Tester trial series. Selection for increasing DEN and PME resulted in a reduction of internal checking. Low heritability in DBH resulted in marginal genetic improvement in DBH and the adverse genetic correlations between DBH, ERB and NHR made it is difficult to reduce incidence of ERB and NHR while keeping increase of DBH in the population. The robust desired-gain selection proposed in this study combines concepts of desired-gain methodology and robust selection. It selected all of individuals by desired-gain index but excluded the individuals with high ranking variability to the change of economic weights. Some NKTs, for example, NHR and ERB, need to be included in selection criteria if breeders want to reduce their incidence in the population. It is concluded that desired-gain index combined with robust selection concept is an efficient way for selecting the key and non-key traits in radiata pine breeding programmes in New Zealand.
